# Supplementary material for: Effects of time management interventions on mental health and wellbeing factors: A protocol for a systematic review
Source: PLoS One. 2024 Mar 11;19(3):e0288887. doi: 10.1371/journal.pone.0288887 (PMC10927119; doi:10.1371/journal.pone.0288887)
Supplement: Attachment — Submitted filename: Response to Reviewers.docx [file pone.0288887.s002.docx]

PONE-D-23-20913

Effects of time management interventions on mental health and wellbeing factors: A protocol for a systematic review

We thank the academic editor and reviewers for their consideration of this manuscript and their thoughtful feedback. Below, we respond to each point raised and how, where applicable, these have influenced revisions made to the manuscript. We believe this feedback and corresponding revisions have served to clarify and strengthen the manuscript and we again thank the academic editor and reviewers for their meaningful contributions to this process.

**Journal Requirements:**

When submitting your revision, we need you to address these additional requirements.

1. Please ensure that your manuscript meets PLOS ONE's style requirements, including those for file naming. The PLOS ONE style templates can be found at

https://journals.plos.org/plosone/s/file?id=wjVg/PLOSOne_formatting_sample_main_body.pdf and

<https://journals.plos.org/plosone/s/file?id=ba62/PLOSOne_formatting_sample_title_authors_affiliations.pdf>.

We apologize for our formatting errors. We have reviewed the manuscript to adhere to formatting guidelines. The tables have been removed as separate file uploads and are only presented within the main manuscript. Supporting information is presented as “S1 Table” in the main manuscript and provided as a separate file upload with correct file naming.

2. Please review your reference list to ensure that it is complete and correct. If you have cited papers that have been retracted, please include the rationale for doing so in the manuscript text, or remove these references and replace them with relevant current references. Any changes to the reference list should be mentioned in the rebuttal letter that accompanies your revised manuscript. If you need to cite a retracted article, indicate the article’s retracted status in the References list and also include a citation and full reference for the retraction notice.

We have reviewed our reference list and confirm that it is complete, correct, and abides by the journal’s formatting guidelines. We have checked all references in the Retraction Watch Database and do not reference any retracted papers in our manuscript.

**Reviewer #1:**

The manuscript is well written with sound methodological considerations for the systematic review. Time management is a challenge for many workers and this tend to affect their mental wellbeing as they are often stressed. Finding effective time management interventions is very critical, and the authors are commended for taking this up. I look forward to the findings of the review.

Thank you very much for this feedback. We are pleased to hear that you resonate with the potential contributions of this work.

Kindly note the spelling of the word "sever" in line 56. Not sure if the intended word is severe.
On line 68, letter 's' may be added to the word intervention, the last word in the line.

These are very detailed catches. Thank you for bringing them to our attention. We have updated the manuscript to reflect these changes.

**Reviewer #2:**

This is a straightforward protocol and will suggest some few points for your consideration:

1. Will it be helpful if the search terms include more specific psychological well-being outcomes such as anxiety etc? Will it change the search results? Please consider further.

Thank you for this consideration. Due to the systematic review’s aim of assessing nonclinical populations (in workplace contexts being exposed to time management interventions), we decided to exclude search terms such as anxiety and depression.

In response to your feedback, we have reviewed the studies included in Aeon et al.’s (2021) extensive meta-analysis of time management. Studies that measured well-being variables (including factors such as anxiety, depression, and psychological distress) were reviewed to see whether they would fit our inclusion criteria. As Aeon et al.’s (2021) meta-analysis should be a comprehensive review of the time management literature, this brief review of their wellbeing-related studies suggests that we have not missed any studies relevant to our review by excluding terms such as anxiety. As a result, we have decided to maintain the original search terms.

2. The data synthesis approach can be sharpened further. For instance, how many authors will be involved in the process, how will conflicting interpretations be resolved? How will the findings be integrated?

Thank you for this feedback. We have provided additional details in the data synthesis section to address these concerns. Details regarding how and which authors will be involved in data synthesis, how conflicting interpretations will be addressed, and how the findings will be integrated have all been included.
